# Drug-induced aortitis of the subclavian artery caused by pegfilgrastim: a case report

**DOI:** 10.1186/s40792-021-01282-9

**Published:** 2021-08-26

**Authors:** Hikari Jimbo, Yoshiya Horimoto, Misato Okazaki, Yumiko Ishizuka, Hidenori Kido, Mitsue Saito

**Affiliations:** 1 Department of Breast Oncology, Juntendo University School of Medicine, 2-1-1 Hongo, Bunkyo-ku, Tokyo, 113-0033 Japan; 2 Department of Medical Oncology, Juntendo University School of Medicine, 2-1-1 Hongo, Bunkyo-ku, Tokyo, 113-0033 Japan

**Keywords:** Breast cancer, Granulocyte-colony stimulating factor, Pegfilgrastim, Drug-induced aortitis

## Abstract

**Background:**

Pegfilgrastim is a modified version of granulocyte-colony stimulating factor (G-CSF), with a polyethylene glycol (PEG) that prolongs its half-life in peripheral blood. It is prophylactically administered during chemotherapy to prevent severe febrile neutropenia. G-CSF-related aortitis is a rare side effect but reports of this disease have been increasing in recent years, probably due to PEGylation. Herein, we report a case who developed pegfilgrastim-induced aortitis, localized to the right subclavian artery, during adjuvant chemotherapy. Her condition recovered without the use of steroids.

**Case presentation:**

A 58-year-old woman was diagnosed with invasive ductal carcinoma of the left breast. She had a medical history of contralateral breast cancer and pyelonephritis. Following curative surgery for her left breast cancer, she received adjuvant chemotherapy. Two days after the first course of dose-dense paclitaxel, pegfilgrastim was used as planned. Eight days after the administration of pegfilgrastim, she developed a high fever of 38 °C and visited the emergency outpatient clinic 3 days after. Blood tests revealed an increased inflammatory response, and contrast-enhanced computed tomography (CT) revealed a wall thickening of the subclavian artery, suggesting aortitis caused by pegfilgrastim. She was hospitalized on day 15 when CRP increased to 21.5 mg/dL and the high fever continued. Blood and urine culture tests were negative throughout. Pegfilgrastim-induced aortitis was suspected and she was observed without the use of steroids. Seven days later, her fever abated. A contrast-enhanced CT scan on day 26 showed the subclavian artery wall thickening had disappeared. The patient continues to be afebrile and is currently on weekly paclitaxel without use of G-CSF.

**Conclusions:**

The onset of this disease is known to usually occur within 2 weeks after the first pegfilgrastim administration. Aortitis localized to the subclavian artery is relatively rare with the most frequent site being the aortic arch. Clinicians should be aware of the timing and location of onset of this disease.

## Background

Granulocyte-colony stimulating factor (G-CSF) binds to its receptors on neutrophil progenitors in the bone marrow and increases neutrophil number in peripheral blood by promoting the differentiation of neutrophil progenitors into neutrophils [1]. It has long been used in chemotherapies that cause myelosuppression. Pegfilgrastim is a modified version of G-CSF, with a polyethylene glycol (PEG) conjugated to the N-terminus of filgrastim to prolong its half-life in peripheral blood [1]. As a sustained form, it can be administered prophylactically during chemotherapy to prevent severe febrile neutropenia. It is widely used for a variety of malignancies.

G-CSF-related aortitis is a rare side effect of G-CSF treatment, with an incidence rate of 0.0014% in the United States and 0.47% in Japan, indicating a slightly higher incidence in Asian patients [2]. It is also known to occur more frequently in women [2, 3]. The key findings for diagnosis are the presence of aortic wall thickening and surrounding soft tissue infiltration on contrast-enhanced CT scan [2–5]. There are no specific markers for G-CSF-associated aortitis and general markers for autoimmune disease, such as PR3-ANCA (proteinase3-anti-neutrophil cytoplasmic antibody), MPO (myeloperoxidase)-ANCA, antinuclear antibodies and IgG, are usually negative [2, 3]. Therefore, C-reactive protein (CRP) is currently used to assess disease activity in clinical practice. Treatment for this disease has not yet been established, as the benefit of steroids in patient remission, in a similar manner to Takayasu’s disease, is equivocal [2–7].

Reports of G-CSF-associated aortitis have been increasing in recent years, probably due to PEGylation [2, 3, 6]. Here, we report a case who developed pegfilgrastim-induced aortitis, localized to the right subclavian artery, during adjuvant chemotherapy. Her condition recovered without the use of steroids.

## Case presentation

A 58-year-old woman found a lump on her left breast and was diagnosed with invasive ductal carcinoma (IDC) by her previous doctor, who referred her to our department for further treatment. Fourteen years prior she had undergone curative surgery for contralateral right breast cancer at another hospital (IDC, triple negative, pT2N0M0). Postoperatively, she received six cycles of CEF (C: cyclophosphamide 500 mg/m^2^, E: epirubicin 75 mg/m^2^, F: 5-fluorouracil: 500 mg/m^2^). She had a history of pyelonephritis. Her family history includes esophageal cancer in her father, cerebral infarction in her mother, and colon cancer in her grandfather.

**Fig. 1:**
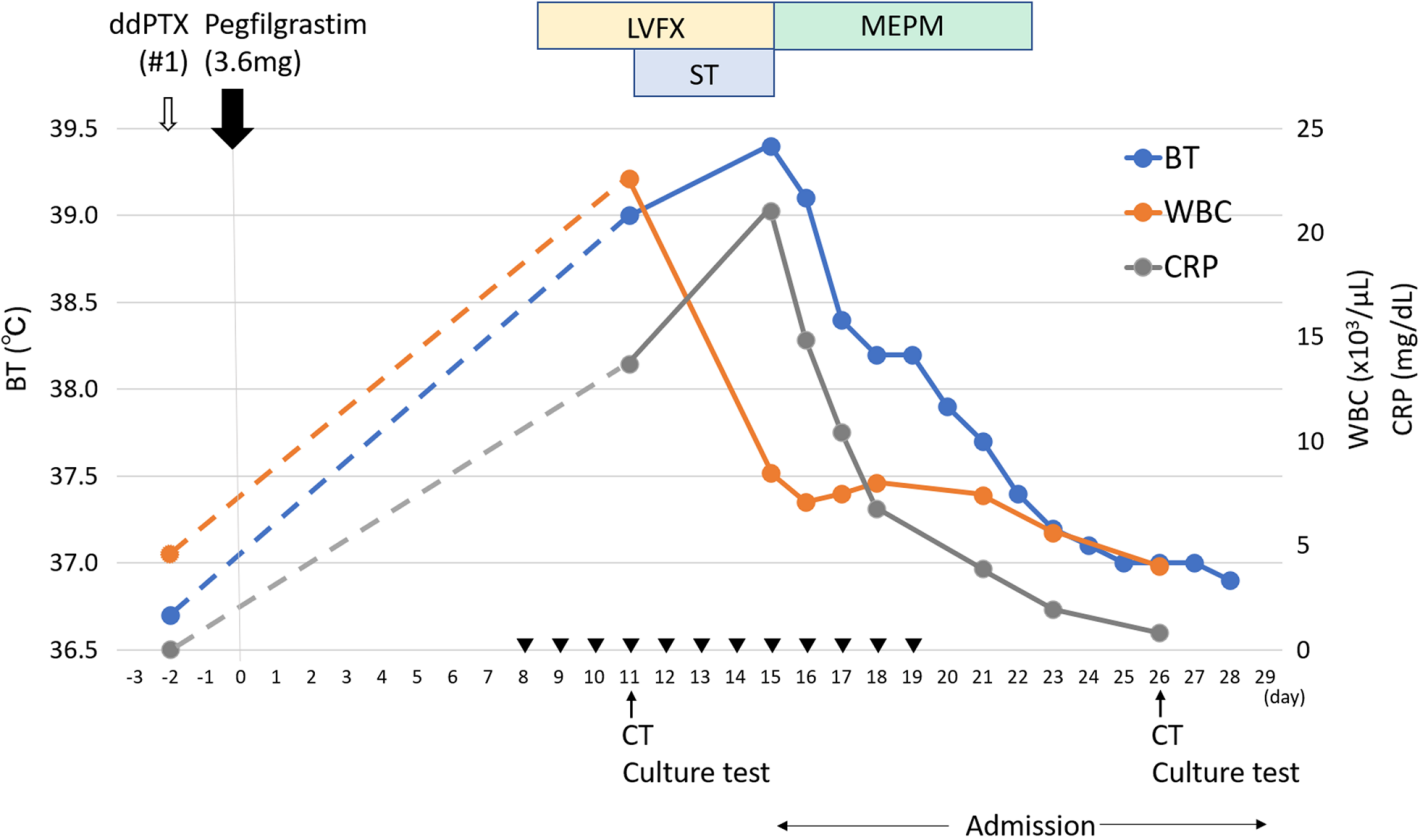
Clinical course of the case patient. The clinical course of this patient after pegfilgrastim administration is shown. Blue, orange and grey lines indicate body temperature (BT), white blood cell (WBC) and CRP, respectively. Down arrowheads indicate the use of acetaminophen. *ddPTX* dose-dense paclitaxel, *LVFX* levofloxacin, *ST* sulfamethoxazole–trimethoprim combination, *MEPM* meropenem, *CT* computed tomography

At our hospital, she underwent left mastectomy and sentinel node biopsy for left breast cancer. The final pathological diagnosis was IDC, ER (−), PgR (+), HER2 (−), pT2N0M0 (stage IIA). After starting TC (docetaxel plus cyclophosphamide) as adjuvant chemotherapy, a rash appeared all over her body. A drug-induced lymphocyte stimulation test (DLST) was positive for docetaxel and palonosetron hydrochloride, so this chemotherapy was discontinued. Instead, she received dose-dense paclitaxel (ddPTX, 175 mg/m^2^ bi-weekly) therapy. Two days after the first course, pegfilgrastim (3.6 mg) was administered as a scheduled regimen. Eight days after the administration of pegfilgrastim, she developed high fever of 38 °C and took levofloxacin (LVFX). As the fever did not decrease within 3 days, the patient visited the emergency outpatient clinic (day 11 after pegfilgrastim administration). Figure 1 shows the clinical course of the patient. Blood tests, contrast-enhanced computed tomography (CT), and various culture tests (blood, urine and sputum) were performed, and an increased inflammatory response was observed with white blood cell (WBC) 22,600/μL (neutrophil: 93%) and CRP 13.7 mg/dL.

**Fig. 2:**
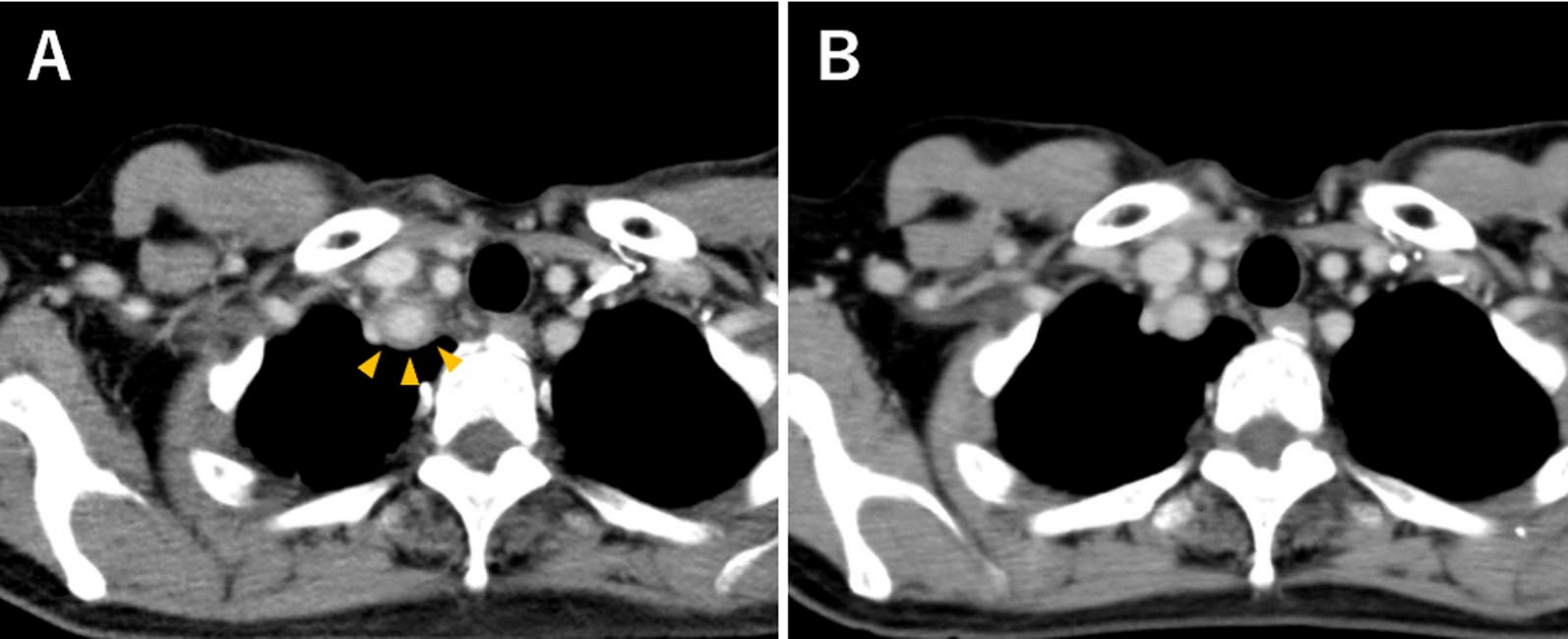
CT scan findings: the right subclavian artery. **A** Wall thickening and peri-aortic soft tissue infiltration of the right subclavian artery on day 11 after pegfilgrastim administration (orange arrowheads). These findings were improved on day 26 (**B**)

Contrast-enhanced CT revealed wall thickening of the subclavian artery, suggesting the possibility of pegfilgrastim-induced aortitis (Fig. 2A). However, pyelonephritis could not be ruled out due to the observation of some poorly contrasted areas in the renal cortex. As a result, she was given an additional sulfamethoxazole–trimethoprim combination, however, the fever did not abate and the patient returned to the outpatient clinic on day 15. At this point CRP had increased to 21.5 mg/dL, and following strong complaints of malaise and anorexia she was hospitalized that day. While blood and urine culture tests were negative, meropenem (MEPM) was given since an anaerobic bacterial infection could not be ruled out. At this point, the possibility of pegfilgrastim-induced aortitis was considered more likely than infection, and the patient was observed without steroids. Acetaminophen was used as needed during admission. On day 21 (7 days after admission) her fever abated, and blood tests showed improvement on day 23 (WBC 5600/μL and CRP 1.95 mg/dL). A contrast-enhanced CT scan was performed again on day 26, and an improvement of the subclavian artery wall thickening was confirmed (Fig. 2B).

Following discharge from hospital, the patient remains afebrile and is currently on weekly paclitaxel without the use of G-CSF.

## Conclusions

This report describes our experience with a case of aortitis localized to the subclavian artery where fever was exhibited 8 days after pegfilgrastim administration. The onset of this disease is known to usually occur within 2 weeks after the first administration of pegfilgrastim [3]. G-CSF-induced aortitis can occur in the thoracic to abdominal aorta and its branches, but the most frequent site is the aortic arch, reported to occur in approximately 70% of cases (11 of 16) [3–12]. Aortitis localized to the subclavian artery is relatively rare.

When our case patient visited an emergency outpatient clinic, her blood test showed high neutrophil rate (93%), despite no infectious disease. In clinical practice, an increase in the WBC neutrophil rate is frequently observed after pegfilgrastim administration. However, to the best of our knowledge, there are no reports on changes in leukocyte fractions after pegfilgrastim administration, but only one old report on conventional G-CSF [13]. For reference, we retrospectively investigated changes in neutrophils after the first administration of pegfilgrastim. We examined patients who received dose-dense EC (E: epirubicin 90 mg/m^2^, C: cyclophosphamide 600 mg/m^2^) as neo-adjuvant therapy during the December 2019 through March 2021 period (*n* = 21), this being the most common regimen for pegfilgrastim use. As shown in Table 1, the neutrophil rate did significantly increase over 2 weeks after the first administration of pegfilgrastim, as well as WBC, absolute count of neutrophils and CRP. Therefore, it is difficult to determine an existence of infection based on a differential white blood count, although the presence of a severe infection may reduce the neutrophil count.

**Table 1:** Changes in neutrophils after the first pegfilgrastim administration (*n* = 21)^a,b^

	Day 0^c,d^	Day 14^c,d^	*P* value
WBC	5395 (2700–8500)	10,838 (5500–19,200)	< 0.001
Neutrophil rate	57.3% (41.8–67.1)	77.2% (59.2–84.2)	< 0.001
Absolute neutrophil count	3126 (1463–5542)	8492 (3300–15,110)	< 0.001
CRP	0.10 (0.03–0.63)	0.37 (0.02–1.45)	0.011

^a^Mean age was 49.8 (range 33–64). ^b^All patients were given dose-dense EC (E: epirubicin 90 mg/m^2^, C: cyclophosphamide 600 mg/m^2^) and pegfilgrastim (3.6 mg) was administered 1–3 days later. ^c^The day of the first dose-dense EC administration was counted as Day 0. ^d^Mean (range)

The treatment for drug-induced arteritis has not yet been established. As to the application of steroids, in cases such as this case where the vasculitis is localized and systemic symptoms are minimal, omission of steroid administration might be possible. However, it is clearly necessary to establish a treatment strategy for this disease by accumulating more cases.

If a patient develops a persistent high fever of unknown origin after pegfilgrastim administration, drug-induced aortitis should be considered during investigations for causes of fever. It is also crucial for clinicians to be aware of the timing and location of onset of this disease.

## Data Availability

Not applicable.

## Abbreviations

ANCA: Anti-neutrophil cytoplasmic antibody
BT: Body temperature
CEF: C: cyclophosphamide, E: epirubicin, F: 5-fluorouracil
CT: Computed tomography
CRP: C-reactive protein
ddPTX: Dose-dense paclitaxel
DLST: Drug-induced lymphocyte stimulation test
G-CSF: Granulocyte-colony stimulating factor
IDC: Invasive ductal carcinoma
LVFX: Levofloxacin
MEPM: Meropenem
MPO: Myeloperoxidase
PEG: Polyethylene glycol
PR3: Proteinase3
ST: Sulfamethoxazole–trimethoprim combination
WBC: White blood cell
